# Humidity stress and its consequences for male pre‐ and post‐copulatory fitness traits in an insect

**DOI:** 10.1002/ece3.10244

**Published:** 2023-07-03

**Authors:** Leigh W. Simmons, Maxine Lovegrove, Xin (Bob) Du, Yonglin Ren, Melissa L. Thomas

**Affiliations:** ^1^ Centre for Evolutionary Biology, School of Biological Sciences The University of Western Australia Perth Western Australia Australia; ^2^ Harry Butler Institute Murdoch University Perth Western Australia Australia; ^3^ CSIRO Health and Biosecurity CSIRO Land and Water Floreat Western Australia Australia

**Keywords:** aridity, climate change, desiccation stress, male fertility, mating behavior, *Teleogryllus oceanicus*

## Abstract

Global declines in insect abundance are of significant concern. While there is evidence that climate change is contributing to insect declines, we know little of the direct mechanisms responsible for these declines. Male fertility is compromised by increasing temperatures, and the thermal limit to fertility has been implicated as an important factor in the response of insects to climate change. However, climate change is affecting both temperature and hydric conditions, and the effects of water availability on male fertility have rarely been considered. Here we exposed male crickets *Teleogryllus oceanicus* to either low or high‐humidity environments while holding temperature constant. We measured water loss and the expression of both pre‐ and postmating reproductive traits. Males exposed to a low‐humidity environment lost more water than males exposed to a high‐humidity environment. A male's cuticular hydrocarbon profile (CHC) did not affect the amount of water lost, and males did not adjust the composition of their CHC profiles in response to hydric conditions. Males exposed to a low‐humidity environment were less likely to produce courtship song or produced songs of low quality. Their spermatophores failed to evacuate and their ejaculates contained sperm of reduced viability. The detrimental effects of low‐humidity on male reproductive traits will compromise male fertility and population persistence. We argue that limits to insect fertility based on temperature alone are likely to underestimate the true effects of climate change on insect persistence and that the explicit incorporation of water regulation into our modeling will yield more accurate predictions of the effects of climate change on insect declines.

## Graphical abstract

The thermal limit to fertility has been implicated as a factor in the response of insects to climate change. However, climate change is also affecting hydric conditions. We demonstrate strong effects of hydration stress on mating behavior and male fertility that will adversely affect insect reproduction under climate change.

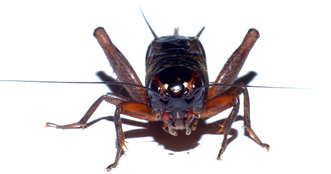

## 1 INTRODUCTION

Global climate change is predicted to drive an unprecedented number of animals and plants to extinction over the coming decades, with some suggesting that the Earth's sixth mass extinction is already underway (Barnosky et al., 2011). Indeed, there is an abundance of evidence that climate change is impacting ecosystems and their constituent species across the Earth's continents and oceans (Parmesan & Yohe, 2003; Root et al., 2003; Rosenzweig et al., 2008).

Insects are the most abundant and diverse animals on Earth (Rosenberg et al., 2023), and they are critical to the functioning of terrestrial and aquatic ecosystems, both natural and agricultural. Insects provide pollination services to flowering plants, they recycle nutrients through decomposition and they underpin the food chain critical to higher organisms, including humans (Harvey et al., 2023; Hazarika & Kalita, 2023; Schowalter et al., 2018; van Huis & Gasco, 2023; Zhou et al., 2023). Alarmingly, insect abundance is declining rapidly. While patterns vary geographically and among taxonomic groups, on average, reported rates of insect decline appear to be in the region of 1%–2% per annum (Wagner et al., 2021). While there are a number of anthropogenic factors that are contributing to this decline, including habitat loss and increased use of pesticides, it is clear that climate change is contributing significantly to the decline in insect populations (Halsch et al., 2021; Janousek et al., 2023). However, we have little knowledge of the direct and indirect mechanism(s) responsible for climate‐induced insect population declines (Wagner et al., 2021), a problem that requires immediate attention (Harvey et al., 2023).

Male fertility is a critical, yet largely overlooked trait in studies of the ecological and evolutionary implications of climate change (Walsh et al., 2019). Being ectothermic, insects are particularly vulnerable to rising temperatures. Like most animals (Breckels & Neff, 2013; Houston et al., 2018; Hurley et al., 2018; Levine et al., 1988; Sales et al., 2018), male insects suffer decreased fertility in response to increased temperature (Baur et al., 2022; Chirault et al., 2015; Gasparini et al., 2018; Martinet et al., 2021; Parratt et al., 2021; Porcelli et al., 2017; Sales et al., 2021). A recent study of flour beetles found that heatwave conditions (5°C above average temperature for >5 days) resulted in a reduction in the ability of males to sire offspring (Sales et al., 2018). Males exposed to heatwave conditions suffered reduced mating behavior, the number of sperm contained within ejaculates, and the viability of sperm, indicating that both premating and postmating sexual traits were negatively impacted by heat stress (Sales et al., 2018). In *Drosophila subobscura* exposure to heat stress reduces sperm motility and subsequent fertility (Porcelli et al., 2017), and for *Drosophila* species generally, males became sterilized on average 1.15°C lower than the Critical Thermal Limit (CTL) at which they perish (Parratt et al., 2021). The Thermal Fertility Limit (TFL), the temperature at which a species loses fertility (Walsh et al., 2019), can more accurately predict the global distribution of *Drosophila* species than does the CTL (Parratt et al., 2021). Indeed, use of the CTL in population modeling was shown to underestimate the potential impacts of global warming on the distribution of *D. flavomontana* by as much as 48% compared with use of the TFL (Parratt et al., 2021). Moreover, the extinction risks of experimentally evolving lines of *Drosophila* species in the laboratory are better predicted by a species' TFL than its CTL (van Heerwaarden & Sgrò, 2021). Given the critical role of fertility in population persistence, thermal limits to insect fertility are likely important contributors to the observed declines in insect abundance.

A considerable body of research has focused on the effects of temperature on species distributions and persistence. This is not surprising, since Global warming is predicted to reach or exceed 1.5°C in the near term (IPCC, 2022), and rising temperature can affect species persistence directly, due to physiological thermal limits (Kearney & Porter, 2009), or indirectly, due to loss of habitat or changes in resource availability and competitors (Dawson et al., 2011; Thomas et al., 2004). Consistent with this prediction, meta‐analyses have indicated significant changes in the geographic distribution of many species, with shifts toward higher, and therefore cooler, latitudes and elevations (Chen et al., 2011). However, these analyses also indicate considerable variation among species in their responses, with 25% of species shifting in their distribution toward lower, and therefore warmer, elevations or latitudes (Moritz & Agudo, 2013). This paradox no doubt stems from our focus on temperature per se. A relatively neglected, yet biologically critical component of climate change revolves around water regulation, especially so for ectothermic species such as insects, reptiles, and amphibians (Rozen‐Rechels et al., 2019). As air temperature increases, so too does its capacity to hold water vapor. The result is a decrease in air “saturation” or Relative Humidity (RH). RH has declined steadily since the 1970s, by as much as 10% globally (Vicente‐Serrano et al., 2018). Decreasing RH increases the vapor pressure deficit of air and exposes animals and plants to increased hydration stress.

Maintaining water balance is a critical physiological function that will influence a species' vulnerability to climate warming (Chown et al., 2011; Ferrín et al., 2023). Indeed, population studies have found effects of both temperature and precipitation on species distributions (Janousek et al., 2023; Kearney et al., 2009, 2018; van Heerwaarden & Sgrò, 2021). Contrary to predictions based on rising temperatures, in California climate change has generated a shift in the distributions of 64 plant species to lower and, therefore, warmer elevations (Crimmins et al., 2011). However, these downhill shifts are readily explained when considering changes in climatic water balance (Crimmins et al., 2011). In their meta‐analysis of changing insect abundance, van Klink et al. (2020) found declines in terrestrial but increases in freshwater insect abundances. These studies all point to the critical importance of water availability for species responses to climate warming. Changes in water availability have the potential to accentuate or buffer the physiological and ecological consequences of climate warming (Cahill et al., 2013). Yet experimental studies that focus specifically on the potential effects of hydration stress on insect mating success (Arya et al., 2021; Gefen & Gibbs, 2009) and fertility (Pérez‐Staples et al., 2018) are rare.

Experimental studies of the effects of climate change on insect fertility typically provide subjects with access to water while manipulating ambient temperature, so that they may underestimate the true effect of climate warming on a species thermal fertility limit. Here we examine the effects of hydration stress on the reproductive potential of male crickets, *Teleogryllus oceanicus* while holding temperature constant. As defined, the thermal limit to fertility is the point at which the qualitative ability of an organism to produce viable offspring under controlled conditions is lost (Walsh et al., 2019). A qualitative loss of the ability to produce offspring could arise because of a male's ability to persuade females to mate during courtship and/or because of his ability to produce and transfer functioning sperm. In *T. oceanicus*, males attract and persuade females to mate via extended periods of courtship, during which females choose among males based on the quality of acoustic (courtship song) and gustatory (cuticular hydrocarbon, CHC) signals (Rebar et al., 2009; Simmons et al., 2013; Thomas & Simmons, 2009b). Moreover, female *T. oceanicus* typically mate with more than one male, resulting in sperm competition among rival males for the fertilization of available eggs (Simmons, 2003; Simmons & Beveridge, 2010). We, therefore, asked how hydration stress affects both pre‐ and postmating male reproductive traits, by assessing the quality of a male's courtship song, the composition of his CHC profile, his ability to produce functioning spermatophores and the quality of sperm contained in the ejaculate. We found strong effects of hydration stress on both pre‐ and postmating reproductive traits, and argue that an explicit consideration of the interacting effects of changing temperature and water availability will be necessary for addressing the effects of climate change on insect declines.

## 2 MATERIALS AND METHODS

### 2.1 Experimental animals

Crickets used in these experiments were drawn from a large outbred stock (>1000 individuals) held at the University of Western Australia, and derived originally from crickets collected from tropical fruit plantations in and around Carnarvon, Western Australia. The stock is reseeded annually with ~50 field‐collected individuals and is housed in a constant temperature room set at 26°C with a relative humidity ~50% and a 12 h:12 h light: dark photoperiod. Male nymphs at the eighth instar were collected from stock culture and held individually in plastic boxes (7 × 7 × 5 cm), provided with cat chow and water ad libitum. Crickets were monitored daily and used in experiments 7–10 days after the molt to adulthood.

### 2.2 Humidity treatments

The lids of individual cricket containers were removed and replaced with fly screen lids to facilitate free airflow between container and the external environment. Water bottles were removed, and crickets in their containers were placed into one of two humidity‐controlled environments. Crickets within their individual containers were placed inside 149‐L storage boxes, on a wire rack that rested above a tray containing a humidifying agent. Once crickets had been stacked inside these boxes, the boxes were sealed with plastic food wrap before securing the lid. High‐humidity was maintained using a saturated solution of potassium chloride. Low‐humidity was maintained using silicon dioxide (silica gel), which was dried in an oven at 60°C and changed every 2 days. Humidity within the storage boxes was recorded periodically throughout the course of the experiment, which lasted for 68 days: the high‐humidity treatment had a mean (±SE) relative humidity of 79.0 ± 0.2% (range 77%–83%) while the low‐humidity treatment had a mean humidity of 26.7 ± 0.3% (range 25%–31%) (*n* = 26). The mean annual 15.00 h relative humidity in Carnarvon, where these crickets were sourced, is 55% (range 52%–59%) (Climate statistics for Australia, sourced June 2023 www.bom.gov.au). The experimental humidities thereby lie 28% below and 24% above the current normal humidities experienced by these insects in their natural population. Crickets were assigned haphazardly to one of the humidity treatments, and were maintained in their humidity treatments for 48 h before being assayed.

### 2.3 Cuticular hydrocarbon profiles

The CHC profile of each male cricket was assayed twice using Solid‐Phase Microextraction (SPME), once before and once after exposure to his respective humidity treatment. Thus, before being placed into their treatments, crickets were weighed and then sedated by placing them into a refrigerator at 4°C for 30 min. Crickets were then rubbed the full length of their bodies with a clean SPME fiber (Agilent) for a total of 2 min at a stroke rate of approximately 1 stroke per second; 60 strokes on the ventral surface and 60 strokes on the dorsal surface. The fiber was then emersed and agitated in 1 mL of Hexane (Merck) to dissolve the CHC sample. Samples of CHC were sealed in Chromacol vials (Agilent) and stored at −20°C until analysis. Fibers were washed twice before reuse, by emersion and agitation for 1 min in each of two beakers containing 50 mL of clean Hexane. Forty‐eight hours after being placed into their respective humidity treatments, crickets were reweighed and their CHCs resampled following the procedures outlined above. Crickets were then frozen, dried in an oven at 60°C for 12 h, and their dry weight and pronotum widths determined.

Hexane extracts were transferred to new Chromacol vials containing 250 μL glass inserts with polymer feet (Insert size: 5.6 × 30 mm, PN:5181–1270, Agilent), the samples dried and then reconstituted to 100 μL. We injected 1 μL of this sample via an Agilent 7693 Autosampler into a gas chromatograph and mass spectrometer (Agilent GCMS 7890B/5977E) operating in the split mode (ratio 2:1) and fitted with an Agilent VF‐WAXms column of 30 m × 0.25 mm internal diameter with 0.5 μm coating (PN: CP9222, Agilent). Helium was used as the carrier gas at a flow rate of 1 mL/min. We optimized separation of the extract using a column temperature profile in which the analysis began at a temperature of 150°C for 1 min and rose to 250°C by 25°C/min for 16 min. Final temperature was set at 255°C for 8 min. The MS transfer line, the ion source, and the quad‐pole temperatures were 280, 230, and 150°C, respectively. The certified saturated alkanes standard (C7–C40, 49452‐U, Merck) was used for concentration calibration and retention index calculation.

Agilent Mass Hunter Software was used to acquire and analyze extracts. These were randomized and analyzed in two independent iterations. The mass spectra of unknown compounds were deconvoluted and identified by NIST MS Search 2.4 and AMDIS_32 with the NIST MS database 2020. The retention index was also used to help identify the compounds. Quantitation of metabolite features, peak detection, deconvolution, filtering, scaling, and integration were all processed by Mass Hunter Quantitative Analysis for GCMS (Ver. 7.045.7).

For statistical analysis, peaks were labeled by peak number, which corresponded to their retention times and mass spectra. We calculated proportional peak area by dividing the area of each peak in a given sample by the sum of all peak areas in that sample. A log contrast transformation was used to remove the problem of nonindependence introduced into the analysis by using proportions (Blows & Allan, 1998). Log contrasts were calculated by dividing an arbitrarily chosen peak (peak 3) by the proportional peak area and then taking the log of the new variables. Log (1 + *x*) was used because not all individuals contained every compound. This transformation has been used previously for hydrocarbon data in *T. oceanicus* (Ng et al., 2018; Simmons et al., 2013; Thomas & Simmons, 2009b, 2010). As in previous studies, we performed a principal component analysis on these new variables following Neems and Butlin (1995).

### 2.4 Courtship song

Courtship song was recorded 48 h after males had been placed into their humidity treatments after they had been weighed for the second time and before the second CHC sampling. A plastic partition (9.5 × 4.6 cm) was placed into each male's container such that the container was divided into two equal‐sized sections. A sexually mature virgin female, 7–14 days post adult eclosion, was placed on the opposite side of the partition to the male who was able to contact the female through an array (5 × 10) of holes in the partition, 5 mm in diameter and spaced 5 mm apart. The pair was left for 2 h or until the male began to court the female, at which time his song was recorded. One minute after the onset of courtship singing, males were recorded for 2 min using a Marantz PMD660 Professional digital recorder via a Røde NTG2 condenser microphone. Sampling frequency was 48 Hz and sampling depth 16 bit. All recordings were made in an anechoic room at 26°C and were saved as WAV files.

**FIGURE 1:**
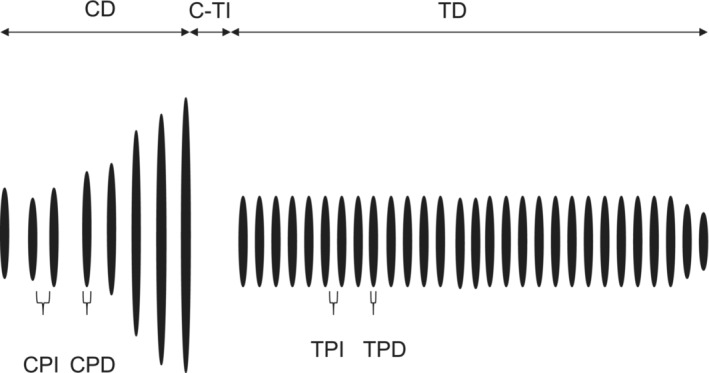
Stylized sonogram of the courtship song of *Teleogryllus oceanicis*. The temporal properties of the song were measured in milliseconds: CD, chirp duration; CPD, chirp pulse duration; CPI, chirp pulse interval; CP#, number of chirp pulses; C‐TI, chirp to trill interval; TD, trill duration; TPD, trill pulse duration; TPI, trill pulse interval; TP# number of pulses in the trill.

Songs were analyzed using Raven 1.6 (Cornell Laboratory of Ornithology). Recordings were filtered to remove noise at <3.5 and >6 kHz. The courtship song has two elements, a chirp followed by a trill (Figure 1). Each element consists of a series of sound pulses each of which correspond to a single wing closure. We measured the duration (ms) of the chirp (CD), the average duration of the last two pulses in the chirp (PD), the average duration of the last two intervals between pulses in the chirp (CPI), counted the total number of pulses in the chirp (CP#), and measured the interval between the chirp and the onset of the trill (C‐TI). We also measured the duration of the trill (TD), the average duration of the fifth and sixth pulses (TPD), the average duration of the sixth and seventh interval between trill pulses (TPI), and counted the total number of pulses within the trill (Figure 1). We measured four consecutive songs per male and took the average parameter values across these songs and entered them into a Principal Components analysis to reduce the number of song variables and obtain major axes of song variation for use in our statistical analyses of the effect of humidity treatment on song quality.

### 2.5 Spermatophore production and ejaculate quality

Immediately after they had been recorded, or after 2 h of exposure to their allocated female if they had not produced courtship song, males were checked for the presence of a spermatophore in the genital pouch by gentle squeezing of the abdomen. Males produce a fresh spermatophore at the start of each scotophase (Loher, 1974) so that spermatophores would have been produced while males were exposed to their allocated experimental environments. If a male had a spermatophore, it was removed with forceps and placed onto a slide with 20 μL of Beadle saline (128.3 mM NaCl, 4.7 mM KCl, and 23 mM CaCl_2_). The evacuating tube was severed with scissors and the ejaculate was allowed to evacuate completely. If the ejaculate did not begin to evacuate within 20 min, the ampulla of the spermatophore was ruptured with forceps to release the ejaculate into the saline. The ejaculate was mixed gently with the saline on the slide. A 5 μL aliquot was then mixed with 5 μL of SYBR 14 (1 mM), diluted 1:50 with Beadle saline and the sample incubated in the dark for 10 min. Then 2 μL of propidium iodide (2.4 mM) was added and incubated in the dark for a further 10 min. The sample was then viewed using a fluorescence microscope, and the number of live (stained green with SYBR 14) and dead (stained red with propidium iodide) sperm in the first 500 sperm counted. The proportion of live sperm in the sample was used as a measure of ejaculate quality.

After checking for spermatophores, males were sedated for their second CHC sampling as described above before being frozen. All statistical analyses were conducted in JMP 15.

## 3 RESULTS

### 3.1 CHC profiles and water loss

We obtained data for a total of 110 males distributed equally across the two humidity treatments (55 high and 55 low). CHC profiles both before and after a male was exposed to its humidity treatment were available for 108 males. We putatively identified 29 hydrocarbon compounds in these CHC profiles that ranged in relative abundance from <0.01% to 26.7% (Table 1). Principal components analysis returned two major axes of variation that each explained >10% of the total variation in CHC profiles. The first axis was weighted positively by compounds with chain lengths C_29_–C_35_ and represented a measure of the relative abundance of all CHC compounds (Table 2). The second PC described qualitative variation in CHC profiles, contrasting the relative abundances of a range of compounds of moderate chain length (C_31_–C_33_) with both shorter (≤C_31_) and longer (C_35_) compounds (Table 1).

**TABLE 1:** Relative abundance of 29 cuticular hydrocarbons identified from 108 male *Teleogryllus oceanicus* sampled before and after they were exposed for 48 h to either a high or low‐humidity environment.

Peak	Putative hydrocarbon	Relative abundance (% ± SD)	PC1 (41.9%)	PC2 (17.4%)
1	Unresolved	0.63 ± 0.76	**0.210**	**0.236**
2	C_29_	0.05 ± 0.11	0.169	0.179
3	Unresolved	0.12 ± 0.12		
4	C_30_	26.77 ± 19.1	**0.226**	0.173
5	x‐meC_31_	0.36 ± 0.18	**0.223**	−0.057
6	Unresolved	0.07 ± 0.06	**0.172**	−0.023
7	C_31:1_	7.88 ± 8.12	**0.187**	**0.202**
8	C_31_	0.02 ± 0.10	0.139	**0.201**
9	C_31:1_	0.00 ± 0.01	**0.203**	**0.233**
10	C_31:1_	2.14 ± 1.00	**0.231**	−0.173
11	C_31:2_	0.10 ± 0.25	0.152	**0.240**
12	C_31:2_	1.66 ± 0.89	0.172	**−0.284**
13	C_31:2_	10.19 ± 5.03	**0.215**	**−0.262**
14	C_31:2_	1.37 ± 0.79	**0.172**	**−0.276**
15	C_32_	0.14 ± 0.19	0.150	0.090
16	x‐meC_33_	0.01 ± 0.02	**0.200**	0.126
17	C_32:2_	0.02 ± 0.02	**0.183**	0.086
18	C_33:1_	0.34 ± 0.23	0.167	−0.198
19	C_33:1_	10.29 ± 10.95	0.154	−0.057
20	C_33_	15.67 ± 13.11	0.122	**−0.199**
21	C_33:1_	0.04 ± 0.04	0.153	−0.016
22	C_33:1_	0.03 ± 0.04	0.164	0.060
23	C_33:2_	1.24 ± 0.54	**0.218**	**−0.218**
24	C_33:2_	5.79 ± 2.66	**0.217**	**−0.214**
25	C_33:2_	14.13 ± 6.33	**0.224**	**−0.247**
26	C_33:2_	0.90 ± 0.40	**0.246**	−0.085
27	C_34_	0.03 ± 0.05	**0.179**	0.076
28	C_35:2_	0.01 ± 0.03	**0.190**	**0.264**
29	C_35:2_	0.01 ± 0.03	**0.186**	**0.275**

*Note*: The contribution of each compound to the first two principal axes of variation (% variance explained) in the hydrocarbon profile are shown (eigenvectors in bold are interpreted as contributing significantly to the axis of variation, being ≥70% of the largest eigenvector, Mardia et al., 1979).

**TABLE 2:** Principal component analysis of the courtship song of males exposed to either a high or low‐humidity environment.

	Mean ± SD	PC1 (29.3%)	PC2 (24.2%)	PC3 (20.1%)	PC4 (10.42%)
CD ms	473.1 ± 86.2	0.269	**0.519**	−0.263	−0.038
CPD ms	35.1 ± 4.5	**−0.498**	0.183	−0.025	0.287
CPI ms	33.8 ± 6.3	0.293	−0.272	0.280	**−0.529**
CP #	7.6 ± 1.5	0.288	**0.496**	−0.314	0.093
C‐TI ms	78.9 ± 29.3	0.054	**−0.362**	0.058	**0.687**
TD ms	2741.7 ± 1054.1	0.138	0.295	**0.602**	0.140
TPD ms	18.7 ± 3.4	**−0.497**	0.140	0.136	−0.317
TPI ms	10.1 ± 2.3	**0.459**	−0.245	−0.125	0.080
TP #	85.5 ± 32.1	0.176	0.281	**0.594**	0.174
High humidity		−0.44 ± 0.20	0.26 ± 0.19	0.21 ± 0.19	−0.11 ± 0.13
Low humidity		1.04 ± 0.31	−0.60 ± 0.30	−0.48 ± 0.29	0.25 ± 0.20

*Note*: The mean song parameters are shown along with the loading coefficients for the first four PCs (eigenvectors in bold are interpreted as contributing significantly to the axis of variation, being ≥70% of the largest eigenvector, Mardia et al., 1979). The mean±SD scores on each PC for males in the high and low humidity treatments are shaded.

Abbreviations: CD, chirp duration; CPD, chirp pulse duration; CPI, chirp pulse interval; CP#, number of chirp pulses; C‐TI, chirp to trill interval; TD, trill duration; TPD, trill pulse duration; TPI, trill pulse interval; TP# number of pulses in the trill (see Figure 1).

**FIGURE 2:**
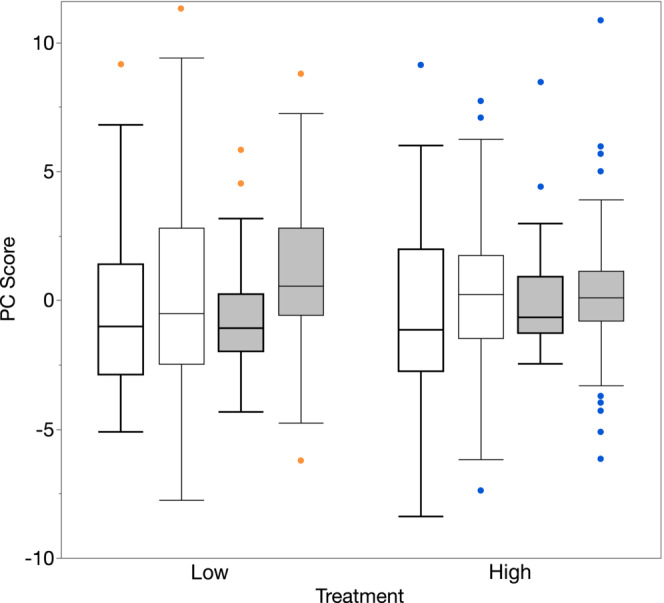
Boxplots of the scores (PC) on the first (open boxes) and second (filled boxes) principal axes of variation in the cuticular hydrocarbon profile for *Teleogryllus oceanicus* sampled before (bold) and after (light) males were exposed to either a low (orange) or high (blue) humidity environment. Boxes represent 50% of the data with the median shown by the horizontal line. Whiskers represent 1.5 times the interquartile range.

Repeated measures ANOVA found no significant effect of treatment on PC1 (*F*_1,106_ = 0.01, *p* = .920) and a marginal within‐subject effect of sampling time (*F*_1,106_ = 3.70, *p* = .057). There was no significant interaction effect between treatment and sampling time (*F*_1,106_ = 0.23, *p* = .630). Males tended to have higher, though not significantly higher, scores on PC1 48 h after the first sampling (Figure 2). For PC2 there was no overall effect of humidity treatment (*F*_1,106_ = 0.52, *p* = .471) and a statistically significant within‐subject effect of sampling time (*F*_1,106_ = 5.54, *p* = .020). There was no significant interaction effect between treatment and sampling time (*F*_1,106_ = 1.91, *p* = .170). Males scored higher on PC2 48 h after the first CHC sample was taken (Figure 2).

**FIGURE 3:**
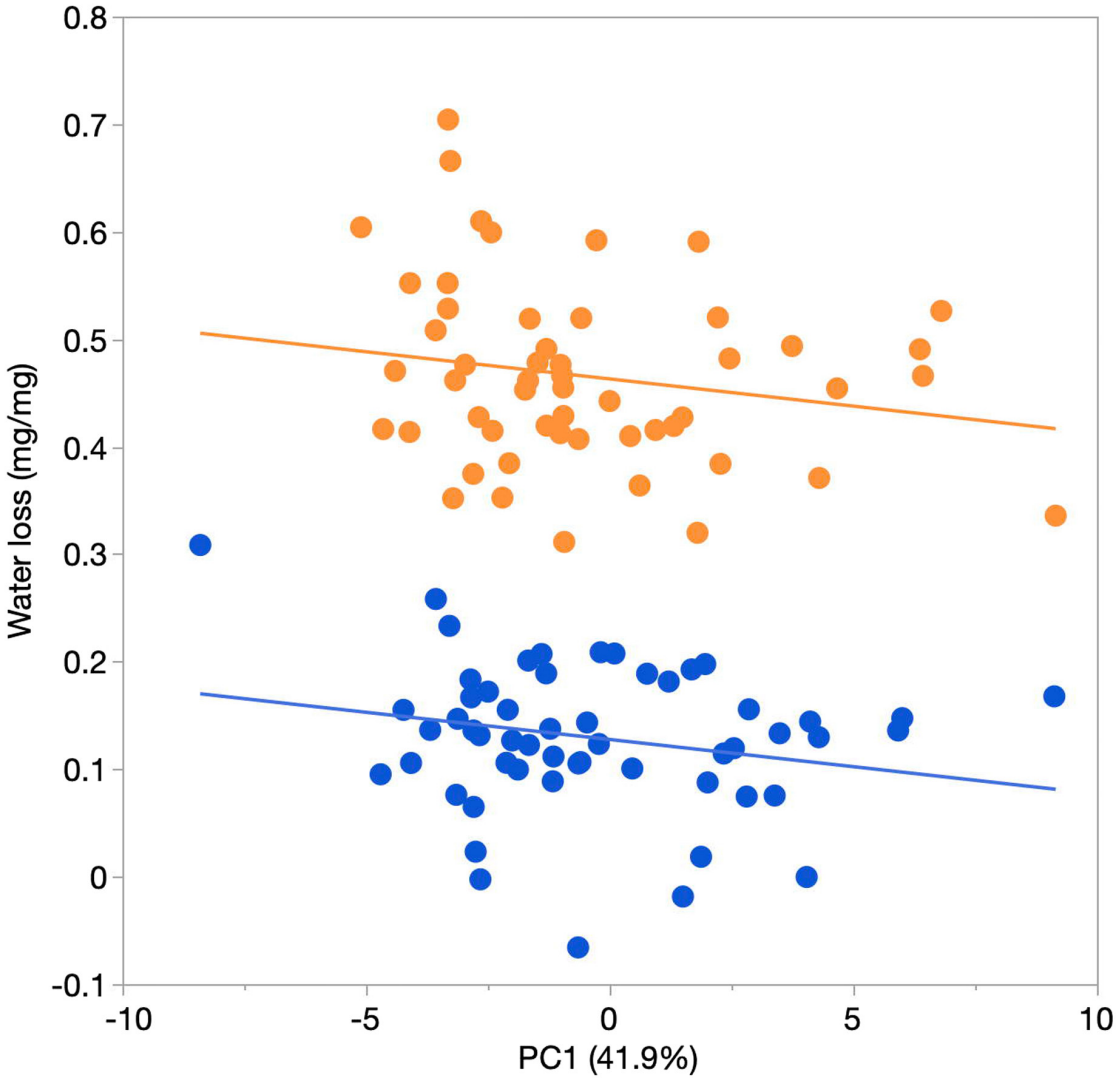
Regressions of water loss (mg per mg dry weight of cricket) on the first principal component (PC1, %variance explained) of variation in the cuticular hydrocarbon profile for male *Teleogryllus oceanicus* held for 48 h either in a low (orange) or high (blue) humidity environment. The CHC data came from analysis of samples collected prior to males being placed into their humidity treatment.

We examined water loss during a male's time spent in the different humidity treatments and the effect of a male's CHC profile on water loss, by entering the change in a male's wet weight over the 48 h period he spent in the humidity treatment, with treatment as the main effect and PC1 (from the pretreatment CHC sample) and dry weight as dependent variables. Heavier males (dry weight) lost more water (*F*_1,103_ = 5.61, *p* = .020) as might be expected. Humidity treatment had a significant effect on water loss (*F*_1,103_ = 636.87, *p* < .001) with males in the low‐humidity environment losing more water than those in the high‐humidity environment (Figure 3). However, there was no significant effect of PC1 on water loss (*F*_1,103_ = 1.73, *p* = .192) and no interaction between PC1 and treatment (*F*_1,103_ = 0.10, *p* = .748). Quantitatively similar patterns were found when using PC2 in the model (Treatment, *F*_1,103_ = 614.54, *p* < .001; PC2, *F*_1,103_ = 0.14, *p* = .710; Treatment × PC2, *F*_1,103_ = 0.01, *p* = .920; dry weight, *F*_1,103_ = 4.50, *p* = .036). Identical results were obtained when PC scores from the post‐treatment CHC sample were used.

### 3.2 Courtship song

Only 49 (89%) males survived to be assayed for courtship song in the low‐humidity treatment, while all 55 (100%) males survived in the high‐humidity treatment (Likelihood ratio *χ* ^2^ = 8.66, df = 1, *p* = .003). Of those surviving, 23 (48.9%) males from the low‐humidity treatment produced courtship song compared with 53 (96.4%) males from the high‐humidity treatment (Likelihood ratio *χ* ^2^ = 33.48, df = 1, *p* < .001).

**FIGURE 4:**
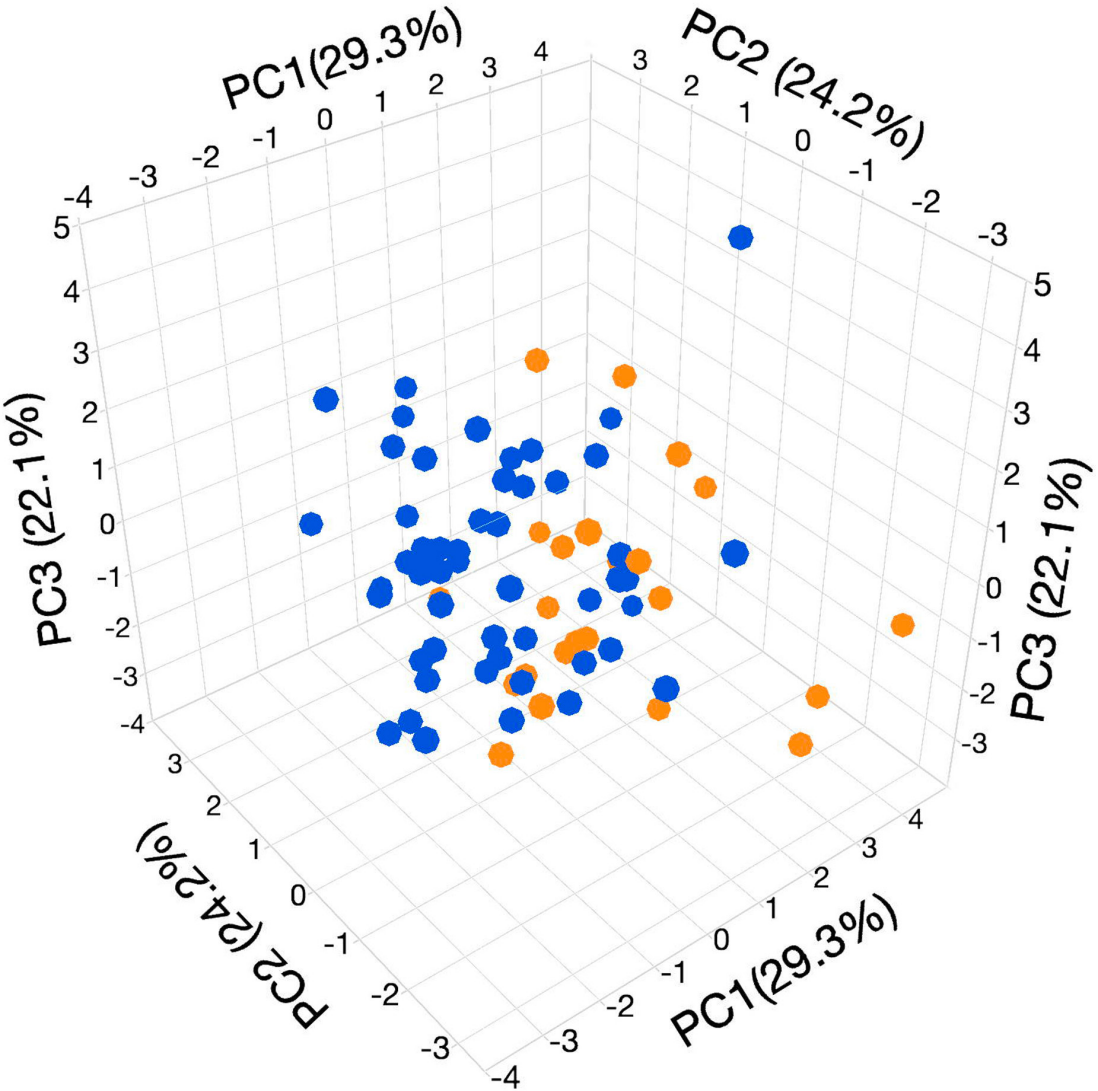
Plot of the scores (PC, % variance explained) on the first three axes of variation in courtship song for male *Teleogryllus oceanicus* held for 48 h either in a low (orange) or high (blue) humidity environment.

Principal components analysis returned 4 axes of variation that each explained >10% of the variation in courtship song structure (Table 2). There was a significant effect of humidity treatment on the first two axes of song variation (PC1, *F*_1,74_ = 16.07, *p* < .001; PC2, *F*_1,74_ = 5.57, *p* = .021; PC3, *F*_1,74_ = 3.85, *p* = .054; PC4, *F*_1,74_ = 2.36, *p* = .129). Males from the low‐humidity treatment produced songs with positive scores on PC1 and negative scores on PC2, while the reverse was true for males from the high‐humidity treatment (Table 2). Thus, compared with high‐humidity males, low‐humidity males produced songs with shorter trills containing fewer, shorter pulses with longer intervals, shorter chirps containing fewer and shorter pulses, and a longer interval between trill and chirp elements (Table 2 and Figure 4).

### 3.3 Spermatophore production and ejaculate quality

**FIGURE 5:**
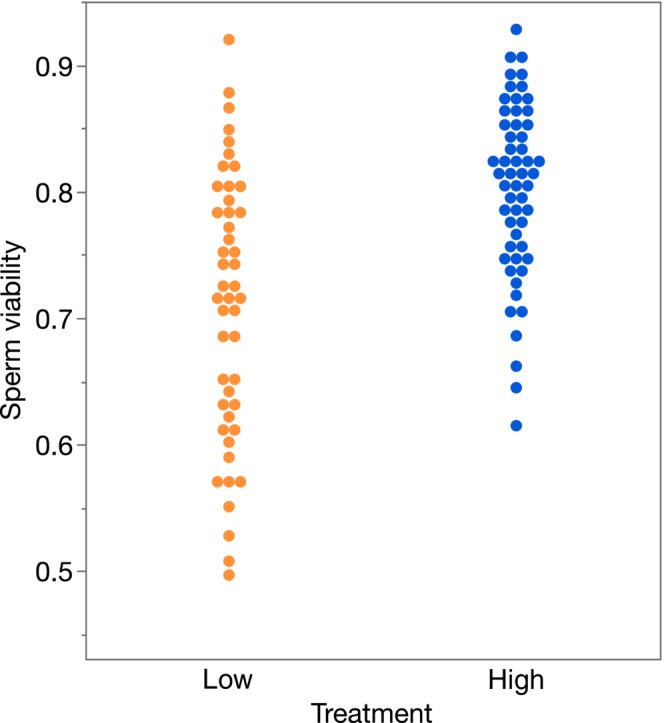
The viability of sperm (proportion alive) contained within the ejaculate of male *Teleogryllus oceanicus* held for 48 h either in a low (orange) or high (blue) humidity environment. The distribution of the data are shown within each treatment.

Of the 49 males that survived the low‐humidity treatment, 47 (95.9%) also produced a spermatophore compared with all 55 (100%) males that survived the high‐humidity treatment (Likelihood ratio *χ* ^2^ = 3.05, df = 1, *p* = .081). Although there was no significant difference in spermatophore production, spermatophore function was affected by humidity treatment. Only 33 (70%) of the spermatophores produced by males from the low‐humidity treatment evacuated their ejaculate compared with 51 (93%) of the spermatophores produced by males from the high‐humidity treatment (Likelihood ratio *χ* ^2^ = 9.14, df = 1, *p* = .003). Moreover, the viability of sperm in the ejaculates of males from the low‐humidity treatment was lower (*F*_1,100_ = 27.86, *p* < .001; Figure 5).

## 4 DISCUSSION

We asked how male reproductive traits, including both premating courtship traits and postmating ejaculate traits, might be affected by hydration stress. Male *T. oceanicus* were housed for 48 h under constant temperature (26°C) with one of two experimentally controlled humidities, either low (RH30%) or high (RH80%). Males from the low‐humidity treatment lost around 30% of their body weight due to water loss, some three times more than males from the high‐humidity treatment. Our manipulation of humidity was, therefore, successful in affecting differences in hydration stress between treatments. The composition of male CHC profiles did not change in response to hydration stress. Males from the low‐humidity treatment were significantly less likely to produce courtship song, and those that did, produced song of lower quality, having a lower duty cycle (sound per unit time) and shorter trill elements. Moreover, although males from the low‐humidity environment were equally likely to produce a spermatophore, their spermatophores were more frequently dysfunctional, failing to ejaculate their contents, and the ejaculate contained a lower proportion of live sperm. The effects of hydration stress on pre‐ and postmating reproductive traits that we have observed are expected to impact directly a male's ability to reproduce.

Hydration stress had a significant impact on the ability of males to court sexually receptive females. Female *T. oceanicus* show strong mating preferences for males able to deliver energetically costly courtship songs, rejecting the courtship attempts of males delivering songs with a low duty cycle; songs that contain few sound pulses particularly in the trill element of the song, and sound pulses that are both short and delivered slowly are unattractive to females (Rebar et al., 2009; Simmons et al., 2013). Only half of the males from our low‐humidity treatment produced a courtship song when presented with a receptive female, and those that did sing produced songs that would have been perceived by females as unattractive. The production of courtship song by male crickets is energetically expensive (Erregger et al., 2017; Hack, 1998; Mowles, 2014), requiring the rapid opening and closing of the modified forewings that are powered by the same musculature used in insect flight (Bentley & Kutsch, 1966). The metabolic energy consumed during singing necessarily demands an elevated exchange of CO_2_ and O_2_ through the tracheal system, and thus increased respiratory water loss through the open spiracles (Chown et al., 2011). One mechanism by which insects can minimize water loss is by reducing their metabolic rate and switching to discontinuous gas exchange (Chown, 2011; Lehmann & Schützner, 2010; Oladipupo et al., 2022). Indeed, in *D. melanogaster*, desiccation stress has been found to have a significant impact on a number of metabolic pathways (Arya et al., 2021). Such physiological response to hydration stress would preclude the production of energetically costly courtship song, and could account for the reluctance of male crickets from our low‐humidity treatment to sing, or to sing highly energetic songs.

Evaporative water loss across the cuticle is perhaps a more significant cause of desiccation stress for insects (Chown & Nicolson, 2004; Quinlan & Hadley, 1993), and the abundance and structure of different cuticular hydrocarbon compounds excreted onto the surface of the cuticle are thought to afford insects protection from excessive water loss (Foley & Telonis‐Scott, 2011; Geiselhardt et al., 2010; Gibbs, 1998). Indeed, for a number of insect species individuals exposed to desiccating environments have been found to change the composition of their CHC profiles to better afford protection from evaporative water loss (Menzel et al., 2018; Sprenger et al., 2018; Stinziano et al., 2015). CHCs are also known to serve important roles in social communication (Blomquist & Bagneres, 2010), and to contribute to male mating success in a number of insect species (Steiger & Stökl, 2014), including *T. oceanicus* (Simmons et al., 2013; Thomas & Simmons, 2009b). The relative strengths of natural and sexual selection acting on insect CHC profiles are not well understood, but CHC blends that best protect individuals from water loss need not be those that contribute most to reproductive success (Berson et al., 2019; Rusuwa et al., 2022). Previously male and female *T. oceanicus* were found to upregulate total CHC production in response to low‐humidity environments experienced during their preadult development (Simmons et al., 2022), suggesting that the quantity of CHCs may be an important defense against desiccation. In the current study we used solid‐phase microextraction techniques to resample the same individuals, so were only able to examine the relative abundances of different compounds, and not the total abundance of each compound. We found no change in the composition of CHC profiles of male *T. oceanicus* in response to the elevated water loss experienced in the low‐humidity treatment. Male *T. oceanicus* have been found to make rapid changes in the composition of their CHC profiles in response to their social environment (Thomas & Simmons, 2009a, 2011), indicating their capacity for short‐term plasticity. Given that we found no significant effect of the CHC composition on the amount of water lost by male *T. oceanicus* in either low or high‐humidity treatments, it may be that adjustments in the relative abundances of different CHC compounds offer little added protection against desiccation stress in this species, which would account for why males did not adjust the relative contributions of different compounds to their CHC profiles in response to the low‐humidity environment. The fact that our humidity treatment had no effect on CHC composition also suggests that, unlike male courtship song, hydration stress may have little qualitative impact on this premating sexual trait. It remains to be seen if this is also the case for total abundance of CHC compounds.

We found that 30% of males from the low‐humidity treatment produced spermatophores that failed to evacuate their ejaculate, so that these males would have experienced complete insemination failure. The cricket spermatophore relies on a mechanism of osmosis to facilitate evacuation (Khalifa, 1949; Sturm, 2003), and as such should be strongly affected by hydration stress. The ejaculate is contained within the cavity of the spermatophore ampulla, surrounded by a crystalline layer that acts as a semipermeable membrane. Outside of the crystalline layer and bound by the outer membrane is a reservoir of evacuating fluid that is unable to pass through the crystalline layer until the reverse pressure across the layer is released by the breaking of the tip of the spermatophore tube when the spermatophore is attached to the females genital opening. Evacuating fluid then passes across the crystalline layer coming into contact with the pressure body, which expands pushing the ejaculate out through the tube and into the female reproductive tract (Khalifa, 1949). Thus, the mechanics of ejaculate transfer rely on the amount and osmolality of evacuating fluid, both of which are expected to depend on water availability. Even if males have the capacity to allocate water reserves for the manufacture of a functional spermatophore, the spermatophores of males housed in a low‐humidity environment may quickly become dysfunctional due to excess water loss when the spermatophore is temporarily held outside of the male's genital pouch while hardening (Loher, 1974). Moreover, the quality of sperm contained within the ampulla of the spermatophore was also affected by our humidity treatments; males in the low‐humidity treatment had ejaculates with lower sperm viability than males from the high‐humidity treatment. The viability of sperm in *T. oceanicus* is affected by the composition of seminal fluid (Simmons & Beveridge, 2011; Simmons & Lovegrove, 2017), which in turn may be influenced by water availability. Indeed, in *Drosophila*, reduced male fertility from simulated heat waves was found in part to arise from damage to the seminal fluid producing accessory glands (Canal Domenech & Fricke, 2022). Given that sperm viability is a major determinant of a male's competitive fertilization success in *T. oceanicus* (García‐González & Simmons, 2005), even if their spermatophores were able to transfer ejaculate to the female, males from the low‐humidity treatment would be expected to have compromised competitive fertility.

An increasing number of studies are revealing the damaging effects of heatwaves on male fertility. Studies are showing that elevated temperatures have detrimental effects on premating sexual signals and on sperm; in parasitoid wasps, heat stress affects spermatogenesis (Chirault et al., 2015), in *Drosophila* it affects sperm motility and fertilization ability (Porcelli et al., 2017), in flour beetles simulated heat waves reduce male mating behavior, sperm numbers and sperm viability (Sales et al., 2018), while in bumblebees they affect male sexual pheromones, the DNA integrity of sperm and their viability (Martinet et al., 2021). Here we show that controlling for temperature, hydration stress associated with decreased humidity has significant detrimental effects on the courtship behavior, sperm transfer and sperm viability of male crickets. We are experiencing both increasing temperatures and changing hydric conditions under climate change, yet the combined effects of thermal and hydric conditions on species vulnerability to climate change have rarely been considered (Chown et al., 2011; Kellermann & van Heerwaarden, 2019; Rozen‐Rechels et al., 2019). For insects, cuticular and respiratory water loss increase rapidly with increasing temperature (Chown, 2002, 2011; Chown et al., 2011) and are predicted to interact in their effects on male fertility. We have realized that thermal limits to fertility are likely significant drivers of insect decline (Walsh et al., 2019), but our current findings for *T. oceanicus* suggest that these limits may be greatly underestimated when based on changes in temperature alone. Incorporating important physiological variables such as water regulation into our modeling will yield more accurate predictions of the effects of climate change on insect species distributions (Bonebrake & Mastrandrea, 2010), and improve our understanding of the mechanisms driving the currant declines in insects globally.

## AUTHOR CONTRIBUTIONS

**Leigh William Simmons:** Conceptualization (lead); data curation (equal); formal analysis (lead); funding acquisition (lead); investigation (lead); methodology (lead); resources (lead); writing – original draft (lead); writing – review and editing (equal). **Maxine Lovegrove:** Data curation (equal); investigation (supporting); writing – review and editing (equal). **Melissa Thomas:** Data curation (supporting); investigation (supporting); methodology (supporting); writing – review and editing (equal). **Xin (Bob) Du:** Data curation (supporting); investigation (supporting); methodology (supporting); writing – review and editing (equal). **Yonglin Ren:** Investigation (supporting); resources (supporting); writing – review and editing (equal).

## CONFLICT OF INTEREST STATEMENT

The authors declare no competing interests.

## Data Availability

Data are available from the Dryad Digital Repository (Simmons et al., 2023) https://doi.org/10.5061/dryad.2ngf1vhv3.
